# The Quality of Response Time Data Inference: A Blinded, Collaborative Assessment of the Validity of Cognitive Models

**DOI:** 10.3758/s13423-017-1417-2

**Published:** 2018-02-15

**Authors:** Gilles Dutilh, Jeffrey Annis, Scott D. Brown, Peter Cassey, Nathan J. Evans, Raoul P. P. P. Grasman, Guy E. Hawkins, Andrew Heathcote, William R. Holmes, Angelos-Miltiadis Krypotos, Colin N. Kupitz, Fábio P. Leite, Veronika Lerche, Yi-Shin Lin, Gordon D. Logan, Thomas J. Palmeri, Jeffrey J. Starns, Jennifer S. Trueblood, Leendert van Maanen, Don van Ravenzwaaij, Joachim Vandekerckhove, Ingmar Visser, Andreas Voss, Corey N. White, Thomas V. Wiecki, Jörg Rieskamp, Chris Donkin

**Affiliations:** 1 University of Basel, Basel, Switzerland; 2 Vanderbilt University, Nashville, USA; 3 University of Newcastle, Callaghan, Australia; 4 University of Amsterdam, Amsterdam, Netherlands; 5 University of Tasmania, Hobart, Australia; 6 Utrecht University, Utrecht, the Netherlands; 7 University of California, Irvine, USA; 8 Ohio State University, Columbus, USA; 9 University of Heidelberg, Heidelberg, Germany; 10 University of Massachusetts Amherst, Amherst, USA; 11 University Groningen, Groningen, Netherlands; 12 Missouri Western State University, St Joseph, USA; 13 Brown University, Providence, USA; 14 University of New South Wales, Sydney, Australia

**Keywords:** Validity, Cognitive modeling, Response Times, Diffusion Model, LBA

## Abstract

Most data analyses rely on models. To complement statistical models, psychologists have developed cognitive models, which translate observed variables into psychologically interesting constructs. Response time models, in particular, assume that response time and accuracy are the observed expression of latent variables including 1) ease of processing, 2) response caution, 3) response bias, and 4) non-decision time. Inferences about these psychological factors, hinge upon the validity of the models’ parameters. Here, we use a blinded, collaborative approach to assess the validity of such model-based inferences. Seventeen teams of researchers analyzed the same 14 data sets. In each of these two-condition data sets, we manipulated properties of participants’ behavior in a two-alternative forced choice task. The contributing teams were blind to the manipulations, and had to infer what aspect of behavior was changed using their method of choice. The contributors chose to employ a variety of models, estimation methods, and inference procedures. Our results show that, although conclusions were similar across different methods, these "modeler’s degrees of freedom" did affect their inferences. Interestingly, many of the simpler approaches yielded as robust and accurate inferences as the more complex methods. We recommend that, in general, cognitive models become a typical analysis tool for response time data. In particular, we argue that the simpler models and procedures are sufficient for standard experimental designs. We finish by outlining situations in which more complicated models and methods may be necessary, and discuss potential pitfalls when interpreting the output from response time models.

## Introduction

In Experimental Psychology, we aim to draw psychologically interesting inferences from observed behavior on experimental tasks. Despite the wide variety of tasks to measure participants’ performance in a range of cognitive domains, many assessments of performance are based on the speed and accuracy with which participants respond. It has long been recognized that the interpretation of data from such response time tasks is hampered by the ubiquitous speed-accuracy trade-off (Pew, 1969; Wickelgren, 1977): When people aim to respond faster, they do so less accurately. Conversely, people can also slow down to increase their accuracy. To understand the implications of this trade-off, consider Mick J and Justin B, 73 and 22 years old respectively. Both perform a simple lexical decision task, where they press as quickly as possible one of two response buttons to indicate whether a string of letters represents a valid word. Now, averaged over many such lexical decisions, Justin turns out to be much quicker than Mick, but Mick has a slightly higher percentage of correct responses. What conclusions should we draw from these results? Is the younger person better at lexical decisions? Is the elderly person more conservative, or maybe just physically slower to press buttons?

To answer such questions, cognitive models have been developed that provide a better understanding of the behavior of participants in response time tasks. These cognitive models are now often used as a measurement tool, translating the speed and accuracy of responses into the latent psychological factors of interest, such as participants’ ability, response bias and the caution with which they respond. In this article, we aim to study the validity of the inferences drawn from cognitive models of response time data. We do so by having 17 teams of response time experts analyze the same 14 real data sets, while being blinded to the manipulations, with the method of their choosing.

In what follows, we begin by introducing the principle of cognitive modeling. Then, we focus on the class of cognitive models most relevant for response time data analysis: evidence-accumulation models. We argue that the validity of inferences from cognitive models are threatened by a host of issues, including those that plague all types of statistical analysis. We then present our collaborative project that we set up to test the validity of the inferences that are drawn using cognitive models for response time data.

### Cognitive Models

The story of Mick J and Justin B is a simple example of the difficulty of a direct interpretation of the observed dependent variables as a measure of performance - being faster or more accurate at a task does not necessarily indicate superior performance. Arguably, ability affects observed data through an intricate series of psychological processes. Cognitive models were developed to provide an explicit account of such psychological processes. A cognitive model is a formalized theory that is intended to mimic the cognitive processes that give rise to the observed behavioral data. Such a formalization often describes a sequence of cognitive steps that are supposedly performed by a participant when performing a task. The precise formalization allows researchers to derive fine-grained predictions about the data that are observed when participants perform tasks that require the targeted cognitive process. Armed with a cognitive model, a researcher can reverse-engineer latent variables of interest from the observed data. For example, she may draw conclusions about participants’ ability from the speed and accuracy of responses.

Perhaps the most-used formal cognitive model of human behavior is signal detection theory (Swets, 2014). Signal detection theory is a mathematical model for the decision over whether a stimulus contains a signal or not. The model is popular because it allows the user to interpret observed responses - the probability of detecting a signal when present and when absent - in terms of psychologically-interesting latent variables, such as the ability to discriminate signal from noise, and the observer’s bias when responding. Similarly, the cognitive models that are the focus of this study, evidence-accumulation models, are used to translate observed response times and choices into the psychologically interesting constructs ease of information processing, response caution, response bias, and the time needed for non-decision processes.

Cognitive models have two key features that explain their current popularity. First, mathematical cognitive models often capture key phenomena in behavioral data for which standard statistical models cannot account. For example, evidence-accumulation models offer a natural account of the relation between response speed and accuracy that is observed in response time experiments. Second, and more crucially for the current study, the parameters of cognitive models reflect the magnitude of assumed cognitive constructs, rather than content-free statistical properties. For example, for a researcher, it is much more interesting to learn that Mick J is twice as good at the task as Justin B, than it is to learn that Justin answers 5% more accurately than Mick, while being on average 150 milliseconds slower.

### Cognitive Models of Response Time Data

In the last few decades, cognitive models have increasingly been used as measurement models for response time data. The most popular of these models is Ratcliff’s (1978) diffusion model. Though originally proposed as an explanation for performance in memory tasks, the diffusion model is now commonly used to transform response time and accuracy data into latent constructs in a wide range of tasks and domains of study. For example, the diffusion model has been used to study implicit racial associations (Klauer, Voss, Schmitz, & Teige-Mocigemba, 2007), effects of aging on brightness discrimination (e.g., Ratcliff, Thapar, & McKoon, 2006), practice effects on lexical decisions (Dutilh, Krypotos, & Wagenmakers, 2011), the effect of attention deficit hyperactivity disorder on a conflict control task (Metin et al., 2013), and the effect of alcohol consumption on movement detection (van Ravenzwaaij, Dutilh, & Wagenmakers, 2012). Response time models have also been applied to inform the analysis of brain measures (e.g., Cavanagh et al., 2011; Forstmann et al., 2008; Mulder, Van Maanen, & Forstmann, 2014; Ratcliff, Philiastides, & Sajda, 2009).

**Fig. 1:**
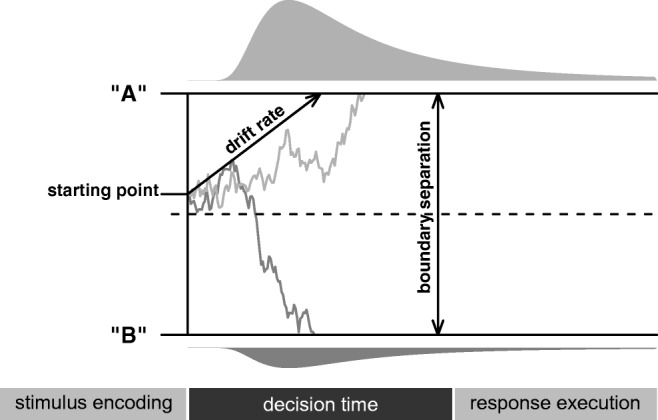
Graphical illustration of the diffusion model

The diffusion model is the prototypical example of an evidence-accumulation model. The model is illustrated in Figure 1. The Figure shows two hypothetical decisions between two options, responses A and B. The accumulation of evidence in favor of either response, as depicted by the solid grey lines, begins closer to the "A" than "B" boundary, indicating a slight bias to respond A. The dark grey line represents a trial on which evidence accumulates relatively quickly towards the "B" boundary, resulting in a fast B response. The light grey line, on the other hand, represents a slow A response. The time to respond is assumed to be the sum of this decision time, plus any time taken to encode the stimuli and execute the motor response (i.e., non-decision time).

#### The Four Key Components of Evidence-Accumulation Models

When used as measurement tools, evidence-accumulation models generally aim to measure four key components. These components are reflected in each of four parameters that are comparable across models.

#### Accumulation Rate

The average rate at which evidence accumulates towards the correct response boundary. The accumulation rate reflects the ratio of signal to noise provided by a stimulus, for a given observer. The accumulation rate parameter is interpreted as quantifying the ease of responding. Faster accumulation rates are associated with quicker and more accurate responding, and slower accumulation rates with slower and less accurate responding. Relatively faster accumulation rates thus indicate participants who are performing better on a task, or stimuli that are easier to process.

#### Boundary Separation

The distance between the two response boundaries in Figure 1. Boundary separation sets the strength of evidence required for either of the response options to be initiated. The boundary separation parameter is interpreted as a measure of response caution. Low boundary separation is associated with quick and error-prone performance, while high boundary separation is associated with relatively slow, conservative performance.

#### Starting Point

The position on the evidence axis at which the information integration process begins. The starting point parameter quantifies the a priori bias towards either of the response options. A starting point that is equidistant from the two boundaries constitutes an unbiased decision process, such that only the sampled information from the stimulus determines the response. A starting point shifted in the direction of one response option (say, option "A" in Figure 1), makes that response occur more frequently and faster, on average. Thus, a shift in starting point signals eventual tendencies to press one button rather than the other, independent of the presented stimulus.

#### Non-Decision Time

The time added to the decision time (i.e., the result of the diffusion process in Figure 1) to yield the observed response time. The non-decision time parameter defines the location of the RT distributions and is assumed to reflect the time that is needed for stimulus encoding and (motor) response execution.

Interpretation of these four key components is the critical factor when using a response time model as a measurement tool. For example, when a researcher aims to study the difference in performance between 72 year old Mick J and 22 year old Justin B, she would fit a response time model to the response time and accuracy data of both participants. The researcher may find that the accumulation rate is slightly higher for Justin, whereas Mick has a larger boundary separation. This result would indicate that the younger participant was better at extracting lexical information from letter strings, but was also less cautious while making his decisions. Thus, instead of interpreting the raw RT and accuracy of both participants, the researcher draws inferences about the latent variables underlying performance, as reflected by the four key components of response time models.

#### Alternative Models for Response Time Data

The diffusion model described above is the most well-known model for response time data. However, there is a range of related models that have been proposed to counter various shortcomings of the model (e.g., Brown & Heathcote, 2005; Kvam, Pleskac, Yu, & Busemeyer, 2015; Usher & McClelland, 2001; Verdonck & Tuerlinckx, 2014). For example, the original "simple" diffusion model does not account for certain typical RT data phenomena, such as the fact that errors are sometimes faster, and other times slower than correct responses. To account for this phenomenon, Ratcliff and Tuerlinckx (2002) introduced the Full Diffusion model, which is more complex than the original diffusion model, assuming trial-to-trial variability in accumulation rate, starting point, and non-decision time (Ratcliff & Tuerlinckx, 2002).

Another difficulty of the diffusion model is that estimating the diffusion model’s parameters is rather complicated. The EZ and EZ2 methods by Wagenmakers et al. (2007) and Grasman, Wagenmakers, and van der Maas (2009) offer simplified algorithms for estimating the diffusion model’s parameters. The simple analytical formula that EZ offers to calculate the diffusion model parameters assumes an unbiased process, and thus prohibits the estimation of a starting point parameter. To address this problem, the EZ2 algorithm was developed to allow the estimation of the starting point parameter.

Similar concerns about estimation complexity led to the development of the Linear Ballistic Accumulation (LBA) model (Brown & Heathcote, 2008). The LBA model is similar to the diffusion model, in that it features the four key components in the diffusion model. However, the LBA differs from the diffusion model in two important ways. First, while the diffusion model assumes that evidence in favor of one response is counted as evidence against the alternative response, the LBA model assumes independent accumulation of evidence for each response. Second, the diffusion model assumes moment-to-moment variability in the accumulation process, while the LBA model makes the simplifying assumption that evidence accumulates ballistically, or without moment-to-moment noise.

In RT data analysis, the various models are generally applied for the same goal: to translate observed RT and accuracy into psychologically interpretable constructs. We think it is fair to say that often researchers base the choice of which model to use on reasons of availability and habit, rather than theoretically or empirically sound arguments. As we argue below, this arbitrariness potentially harms the validity of the results of RT modeling studies.

### Threats to the Validity of Cognitive Models

#### Researcher Degrees of Freedom

Cognitive models suffer from many of the same issues that burden any statistical analysis. For example, in recent years it has become widely acknowledged that most statistical analyses offer the researcher an excess of choices. These large and small choices lead the researcher into a "garden of forking paths" (Gelman & Loken, 2013), where, without a preregistered analysis plan, it is extremely difficult to prevent being biased by the outcomes of different choices. For example, Silberzahn et al. (2015) used a collaborative approach, similar to the approach we take, to demonstrate the degree to which researcher choices can influence the conclusions drawn from data. Their results, as well as many others, have shown repeatedly that such researcher degrees of freedom can threaten the validity of the conclusions drawn from standard statistical analyses. In this light, the expanded landscape of RT models described above might actually provide a threat to the validity of conclusions drawn based on models for RT data analysis. It is not clear to what extent the choice of a certain RT model influences which conclusions one draws.

On top of this, once a model is selected, researchers still face a number of choices regarding their analysis of response time data. For example, one must choose a method for estimating parameters from data. Though Frequentist approaches, such as chi-square (Ratcliff & Tuerlinckx, 2002), maximum-likelihood (Donkin, Brown, & Heathcote, 2011a; Vandekerckhove & Tuerlinckx, 2007), or Kolmogorov-Smirnov (Voss & Voss, 2007) are and remain commonplace, Bayesian methods are becoming increasingly popular (Vandekerckhove, Tuerlinckx, & Lee, 2011; Wabersich & Vandekerckhove, 2014; Wiecki, Sofer, & Frank, 2013). Beyond estimation, there still also remains a choice over how statistical inference will be performed. Historically, null-hypothesis significance tests on estimated parameters were standard (Ratcliff, Thapar, & McKoon, 2004). However, based on growing skepticism over such techniques (e.g., Wagenmakers, 2007), model-selection based methods (Donkin Donkin, Brown, Heathcote, & Wagenmakers, 2011b), and inference based on posterior distributions is now common (Cavanagh et al., 2011).

In summary, there are a very large number of modeler’s degrees of freedom. Further, it is rare to see any strong motivation or justification for the particular choices made by different researchers. The extent to which the various factors influence the conclusions drawn from an evidence-accumulation model analysis is under-explored (but see Donkin, Brown, & Heathcote, 2011a; Lerche, Voss, & Nagler, 2016; van Ravenzwaaij & Oberauer, 2009).

#### Convergent and Discriminant Validity

We have argued that the key benefit of a cognitive model over a statistical model is the ability to make inferences about psychologically interesting latent variables. However, such an advantage relies on the parameters of the cognitive model being valid measures of their respective constructs. We must assume both convergent and discriminant validity, such that a manipulation of response caution, for example, should always affect boundary separation and boundary separation only. There exist already a number of studies which aimed to validate the inferences drawn from evidence accumulation models. We summarize their results and argue why the current large-scale validation study fills a timely place in the literature.

Voss, Rothermund, and Voss (2004) provided one of the earliest systematic empirical validation studies. In their study, participants judged the color of squares made up of green and orange pixels. Crucially, their study manipulated 4 factors across different blocks: the difficulty of the stimuli, participants’ response caution, the probability of green vs orange stimuli, and the ease with which the motor response could be made. After fitting the Full diffusion model, Voss et al. (2004) observed evidence for the convergent validity of the interpretation of the model’s parameters; for example, easier stimuli led to higher estimates of the accumulation rate parameter. Although the experimental manipulations influenced the intended parameters, the effects were not exclusive, speaking against the discriminant validity of the interpretation of diffusion model results. For example, increased caution was not only expressed in higher boundary separation, but also in larger non-decision time estimates.

Other empirical validation studies provide mixed support for convergent and discriminant validity of the Full Diffusion model. On the one hand, Ratcliff (2002) used a brightness discrimination task to demonstrate both forms of validity in accumulation rate, starting point, and boundary separation parameters. However, in a recent study, Arnold, Bröder, and Bayen (2015) found evidence for convergent, but not discriminant validity of the Full Diffusion model parameters in the domain of recognition memory. For example, they found that their manipulation of response caution had a large effect on the boundary separation parameter, but also on the accumulation rate and non-decision time parameters. It is important to note here that conclusions about divergent validity rely on the assumption of selective influence of the manipulations in a validation experiment: for example, a manipulation of response caution should affect response caution, and nothing else. In our results and discussion sections, we will cover this issue in more depth. Though the majority of empirical validation studies have used only the Full Diffusion model (Ratcliff & Tuerlinckx, 2002), some work has also tried to validate alternative response time models. For example, Arnold et al. (2015) found that the EZ diffusion model, unlike the Full Diffusion model, showed both convergent and discriminant validity. Donkin, Brown, Heathcote, and Wagenmakers (2011) re-analyzed the lexical decision data set from Wagenmakers, Ratcliff, Gomez, and McKoon (2008) using both the Full Diffusion and LBA models. With both models, they found convergent and discriminant validity. A similar re-analysis of the data set from Ratcliff et al. (2004) also indicated that the LBA and diffusion models yielded the same conclusion about the influence of aging on recognition memory tasks.

### A Collaborative, Blinded Validation Study

Given the wide-spread application of evidence accumulation time models, the number of attempts to validate the inferences drawn from these models is small. Also, the results from existing validation studies are mixed. Further, we know of no attempt to investigate the extent and influence of researcher degrees of freedom in the application of cognitive modeling. We attempted to address these issues with a large-scale collaborative project.

In our study, we created 14 two-condition data sets and openly invited a range of modeling experts to analyze those data sets. 17 teams of experts committed to contribute. The contributors were asked to infer the difference between the two conditions in terms of four psychologically-interesting latent constructs - the ease of processing, caution, bias, and non-decision time. The 14 data sets, comprising human data, were created such that the two conditions could differ in terms of any combination of ease, caution, and bias. We did not manipulate the non-decision time because the component is not related to a clearly defined construct, but simply reflects the amount of time added to the decision process to yield a response time.^1^ Our collaborative approach offers two advantages over existing validation studies.

First, this approach allows us to assess the validity of a range of methods and models. In particular, since the expert collaborators chose their own methods to draw inferences, we have a sample of the validity of currently popular methods. This range of methods allows us to test the importance of the many choices that response time modelers face when analyzing response time data. This aspect of the project is critical, because most existing validation studies used just one method of analysis. For example, despite their comprehensive analysis, Voss et al. (2004) could draw conclusions about only the Full Diffusion model with Kolmogorov-Smirnov estimation performing null hypothesis tests on estimated parameters. Second, the expert contributors to our study performed their analysis while "blinded". That is, the experts did not know the true manipulations that we made in the validation data sets. The use of this blinded analysis provides a strong advantage relative to existing validation studies. For example, in Donkin, Brown, Heathcote, and Wagenmakers (2011), the authors re-analyzed a data set that had already been analyzed with a Full Diffusion model. As such, Donkin et al. knew the conclusions drawn by the original authors, and so may have made choices that were biased to draw inferences consistent with previously published results. The model-based analysis of response time data is like any other data analysis, and there are many choices on offer to the researcher. Gelman and Loken (2013) argue that without pre-registration, such choices tend to be made so as to increase the likelihood of agreeable conclusions. Our blinded method guards against such potential biases. Indeed, a blinded analysis seems critical in an assessment of the impact of researcher degrees of freedom.

## The Experiment

To create the 14 data sets that the teams of contributors analyzed, we collected data in a factorial design, in which we aimed to impose 1) two levels of difficulty (hard and easy trials) crossed with 2) two levels of response caution (speed and accuracy emphasis instructions), and 3) three levels of response bias (proportion of trials of each response type). Response caution and bias were manipulated across blocks of trials, whereas difficulty of the stimuli was manipulated within blocks. In this section, we describe the experiment, and show that the manipulations had their intended effects on behavior.

### Method

#### Participants

Twenty psychology students (15 female, mean age 26.7, SD = 2.1) at the University of Basel participated in a single session for course credit. The entire session lasted slightly less than two hours. Participants were allowed to take a break between blocks (see below).

#### Materials

Participants performed a random dot motion task which was presented using the Psychopy package for Python (Peirce, 2007). We chose to use the random dot motion task for two reasons: 1) It is a popular task, and we hope that our results can be reasonably generalized to many other simple decision-making tasks. 2) The RDM task permits the fine-tuning of the difficulty of trials, allowing us to easily collect data with a desired number of errors in all cells of the design. In the random dot motion task, participants detect the direction (left or right) of the apparent motion constituted by a cloud of moving dots. Each stimulus consisted of 120 dots (each dot was 4 pixels wide), presented in a circular aperture (diameter = 400 pixels) on a 1680 × 1050, 60 Hz LCD screen. On the first frame of a trial, all dots were placed on random coordinates in the aperture. Then, for each subsequent frame, the dots were displaced according to the following rules. For the difficult stimuli, 10% of the dots moved 1 pixel every 6 frames in the target direction. The other 90% of dots were replaced randomly in the aperture. For the easy stimuli, 20% of the dots moved coherently in the target direction. Coherently moving dots traveled a maximum of 5 pixels, so that no single dot could be monitored to infer the correct direction of motion. Each stimulus had a maximum duration of three seconds. The interval between a response and the start of the next trial was a draw from a uniform distribution between 0.5 and 1 second.

Responses were registered as button presses on a computer mouse with high timing accuracy (1000 Hz). The left and right responses were given by pressing the right hand index and middle finger respectively. Participants were seated in front of a computer screen in a small room in the presence of an experimenter.

#### Design

The experiment used three manipulations.

#### Response Caution (Speed/Accuracy Instructions)

In about half of the blocks, accuracy was emphasized (ac in Table 1). In these blocks, participants received a feedback message "error" on erroneous responses and no feedback on speed, except for "time is up" after three seconds. In the other half of the blocks, speed was emphasized (sp in Table 1). In these blocks, participants received feedback only on the speed of their response: Responses slower than 0.8 seconds resulted in a "too slow" message. No feedback on accuracy was given. All feedback messages lasted 1.5 seconds.

**Table 1:** Manipulations of response caution and response bias across blocks and descriptive statistics per block

Block	1	2	3	4	5	6	7	8	9	10	11	12	13	14	15	16	17	18
Speed–accuracy	sp	ac	sp	sp	ac	ac	sp	sp	ac	ac	sp	sp	ac	ac	sp	sp	ac	sp
bias	no	no	no	left	left	no	no	right	right	no	no	left	left	no	no	right	right	no
RT (ms)																		
.1 quantile	360	510	370	380	490	490	360	380	470	480	370	370	490	490	370	380	480	370
.5 quantile	490	670	490	500	640	640	470	490	610	610	480	480	630	630	470	480	610	480
.9 quantile	690	1040	660	670	970	990	640	660	920	900	660	630	960	980	630	650	910	650
accuracy	0.76	0.91	0.82	0.8	0.91	0.91	0.8	0.84	0.91	0.91	0.81	0.8	0.91	0.9	0.78	0.81	0.91	0.79

Top section: the design of the experimental blocks in the experiment. Bottom section:

Descriptive statistics for each experimental block. Note that behavior remains largely invariant over the course of the experiment. Response caution (speed: sp vs. accuracy: ac) and response bias were manipulated across blocks. Difficulty was manipulated within blocks

#### Bias

In about half of the blocks, left and right stimuli occurred equally often. In the rest of the blocks, stimuli in one direction occurred twice as often (2/3) as stimuli in the other direction (1/3). For one half of these biased blocks, the left stimulus occurred more often, for the other half, the right stimulus occurred more often.

#### Ease/Difficulty

Each block consisted of 50% easy and 50% hard trials, randomly intermixed. Difficulty was manipulated within the blocks to rule out the possibility that participants adjust their response caution when difficulty changes.

#### Procedure

Trials were administered in blocks. The experiment started with 5 practice blocks of 80 trials each, totaling 400 trials. These practice blocks familiarized the participants both with the stimuli (presenting slightly easier stimuli in the first block) and the manipulations of response caution and bias. The main experiment consisted of 18 blocks consisting of 156 trials each (see Table 1).^2^

Before each block, instructions were shown concerning response caution and bias in the upcoming block. This screen informed the participant to either focus on accuracy or speed and the relative number of left and right stimuli in the upcoming block. For non-biased blocks, participants were informed that the relative frequency of left and right stimuli was 50-50; for biased blocks, participants were informed that one direction was twice as likely to occur as the other. It was stressed that this information was accurate and could thus be used to inform choices.

As can be seen in Table 1, from block 2 through block 17, each cell resulting from the factors *response caution × response bias* occurs 4 times over the experimental session. The first and 18^th^ block contain two further blocks in which speed was emphasized and no response bias was implemented. In this cell of the factorial design, more trials were needed, as we explain below.

### Behavioral Manipulation Checks

Naturally, the experimental manipulations were intended to have effects on participants’ overt behavior. Indeed, the speed/accuracy emphasis instruction and difficulty manipulations had the expected effects on the participants’ accuracy and response time. Looking first at proportion correct, we see that participants were more accurate in the accuracy-emphasis condition (${\widehat{P}}_C=0.95$ correct, *SD* = 0.04) than the speed-emphasis condition (${\widehat{P}}_C=0.85$, *SD* = 0.11) when the task was easy. The same pattern was observed when the task was harder, where participants were more accurate under accuracy emphasis (${\widehat{P}}_C=0.86$, *SD* = 0.07) than speed emphasis (${\widehat{P}}_C=0.75$, *SD* = 0.10). A 2 (Emphasis Instruction: Speed vs. Accuracy) × 2 (Difficulty: Easy vs. Hard) × 3 (Bias: Left, None, Right) Bayesian ANOVA on the arcsine-transformed proportion of correct responses confirmed these observations.^3^

The data were best explained by a model with the two main effects of difficulty (*ω*^2^ = 0.95) and emphasis instruction (*ω*^2^ = 0.63), with a BF = 2.11 × 10^24^compared to the null, intercept-only model. There was moderate evidence for the two main effects model over a model that also contained the interaction between difficulty and emphasis instruction (BF = 4.6). The two main effects model was also favored over a model that also included a main effect of the bias manipulation on overall accuracy (BF = 7.45).

The difficulty and emphasis instruction manipulations also influenced the speed of responding. When asked to respond relatively quickly, participants were only a little faster when the task was easy $\widehat{R}T=0.49s, SD=0.05$ compared to when the task was difficult ($\widehat{R}T=0.52s, SD=0.05$). However, when asked to be accurate, participants were much faster in the easy task ($\widehat{R}T=0.63s, SD=0.11$) than in the hard task ($\widehat{R}T=0.75s, SD=0.15$). The same 2 × 2 × 3 Bayesian ANOVA, but on the mean response times, confirmed the apparent interaction between difficulty and emphasis instruction. The model with both main effects, difficulty (*ω*^2^ = 0.29) and instruction (*ω*^2^ = 0.70), and their interaction (*ω*^2^ = 0.75), was the best model (BF = 3.11 × 10^32^ against the null model). There was strong evidence for the model with an interaction term over the model without an interaction between difficulty and emphasis instruction (BF = 14.11 in favor of the model with an interaction). Again, there was little evidence that the bias manipulation had any influence on the overall speed of responding. For example, adding the main effect of a bias manipulation to the best-fitting model reduces the evidence for the model (BF = 14.36 in favor of the model without a main effect of bias).

We use a signal detection analysis to determine whether the bias manipulation had its intended effect. We calculate the standard criterion value, *C*, using the equation 0.5(Φ(*H*) + Φ(*FA*)) where *H* and *FA* are the proportion of ‘left’ responses to leftward and rightward moving dots, respectively, and Φ is the cumulative distribution function of a standard normal distribution. When emphasizing speed, we see a clear effect of the bias manipulation (leftward bias: $\widehat{C}=0.14$; no bias: $\widehat{C}=-0.025$; rightward bias: $\widehat{C}=-0.26$). When participants were instructed to be accurate, however, there was no systematic influence of the bias manipulation (leftward bias: $\widehat{C}=-0.10$); no bias: $\widehat{C}=-0.13$; rightward bias: $\widehat{C}=-0.10$). A 2 × 2 × 3 Bayesian ANOVA on the criterion values indicates that a model with main effects of bias (*ω*^2^ = 0.47) and emphasis instruction (*ω*^2^ = 0.20) and their interaction (*ω*^2^ = 0.75) provides the best account of our data (BF = 3.1 × 10^8^ compared to the intercept-only model). The difficulty manipulation appeared to have no influence on the level of bias shown by participants (BF = 4.84 in favor of the best fitting model, relative to a model that also included a main effect of difficulty).

These analyses show that all our manipulations effected the participants’ behavior to a measurable degree. This is important to know, because we now know there are effects on the overt behavior about which the response time models should in principle allow us to draw inferences. The only condition in which it is unclear whether there was a behavioral effect was the bias manipulation while accuracy was emphasized. We also note that these behavioral manipulation checks are flawed, since response time and accuracy are treated separately, and the analysis permits no inferences about latent factors such as caution and ability. Of course, such critiques are precisely the reason that response time models were proposed in the first place.

## The Creation of the 14 Data Sets

We used the data from the factorial experiment described above to construct the 14 data sets that we asked the contributors to analyze. Each data set had two conditions (A and B), which differed on a different combination of effects, as shown in Table 2. The letters in the table indicate which of the two conditions has a higher value on the relevant component, "-" indicating no difference. Effects are defined as 1) ease: higher means easier, 2) caution: higher means more cautious, 3) bias to right: higher means stronger bias towards the right response option. These 14 data sets constitute the complete set of possible combinations of directions of effects.

**Table 2:** Pseudo experiments

exp	ease	caution	Bias R	Ndt	blocks cond. A	blocks cond. B
1	–	–	–	–	hard, speed, no bias	hard, speed, no bias
2	B	–	–	–	hard, speed, no bias	easy, speed, no bias
3	–	B	–	–	hard, speed, no bias	hard, accuracy, no bias
4	–	–	B	–	hard, speed, no bias	hard, speed, bias
5	B	B	–	–	hard, speed, no bias	easy, accuracy, no bias
6	B	–	B	–	hard, speed, no bias	easy, speed, bias
7	–	B	B	–	hard, speed, no bias	hard, accuracy, bias
8	A	B	–	–	easy, speed, no bias	hard, accuracy, no bias
9	A	–	B	–	easy, speed, no bias	hard, speed, bias
10	–	A	B	–	hard, accuracy, no	hard, speed, bias
11	A	B	B	–	easy, speed no bias	hard, accuracy, bias
12	B	A	B	–	hard, accuracy, no	easy, speed, bias
13	B	B	A	–	hard, speed, bias	easy, accuracy, no bias
14	B	B	B	–	hard, speed, no bias	easy, accuracy, bias

Each line shows for one data set which of the two conditions (A or B) was manipulated to have a higher value on each of the components: ease, caution, bias toward Response B and nondecision time. "-" indicates no difference. Rightmost columns show from which conditions (see Table 1) the data in each of the two conditions originate

### Relabeling of Left and Right

Note that we manipulated bias both favoring the right option (blocks 8, 9, 16, 17) and left option (blocks 4, 5, 12, 13). By doing so, we were able to create a balanced design for the participants: overall, the correct response was equally often left and right. Before constructing the data sets, however, we recoded the data such that the option favored by the bias manipulation was always labeled "right". For all blocks without a bias manipulation, we also flipped the labeling of both stimulus direction and response for the even half of the trials. We did so to ensure that any natural bias of participants to press left or right was averaged out in the data. The analysts contributing to this study were not aware of any re-labeling. However, we did note in the description of the data set: "The coding of left and right stimuli and responses has in some cases been recoded to obfuscate the study’s design. Practically, however, L and R [labels in the data set] should be treated simply as left and right."

### Selection of Cells to Construct Pairs

For many of the combinations of effects shown in Table 2, more than one pair of cells from the factorial design could be chosen. For example, to create two sets that differ on only response caution, one could contrast condition A: easy, accuracy stress, no bias against condition B: easy, speed stress, no bias. One could, however, also contrast condition A: difficult, accuracy stress, bias against condition B: difficult, speed stress, bias. In all such situations where more than one pair could be constructed, we chose the pair with 1) no bias and 2) the highest expected number of errors. The latter condition was chosen to anticipate that many of the models applied to RT data rely on the presence of error responses.

The trials constituting the conditions in each data set were random draws from the relevant cells of the factorial design. The sampled trials could come from all blocks across the experiment, potentially raising issues with non-stationary data (e.g., due to practice or fatigue effects). Table 1 reports the speed of responding and overall accuracy for each block in the experiment. At the aggregate level, the speed and accuracy of participants’ responses remain stable across the entire experiment. In an online supplementary material, we report a more comprehensive demonstration that our decision to sample trials across blocks of a two-hour experiment did not compromise our ability to judge the accuracy of the inferences made by our modeling teams.

Note that Data Set 1 is a special case where there is no difference between the two conditions. To construct this data set, we needed two replications of the same cell in the design. Following the rules above, we chose the cell difficult, speed stress, no bias to constitute both conditions here. Because this cell should contain enough data to be split into two conditions, we needed to collect more data in this cell, hence the addition of block 1 and 18 to the testing session.^4^

### Order of Data Sets, Conditions, and Participants

To obfuscate the design of the study, we shuffled the order of the 14 data sets before we shared them with the modeling experts. Furthermore, the order of conditions A and B was flipped for half of the data sets.^5^ For each data set, the participant numbers were shuffled, and the modelers were made aware that participant numbers were not consistent across the 14 data sets.

### Invitation of Contributors

A large number of response time data analysis experts were invited via email. The invitation letter was and is still available on the open science framework page of this project (https://osf.io/9v5gr/). The invitation letter contained a description of the goal of the project and a very limited description of the method. We carefully wrote this invitation such that the contributors could only know that 1) the data sets comprised real data, 2) the task was a random dot motion task, 3) each two-condition experiment should be treated as a separate data set, and 4) the order of trials was shuffled for all participants, so that sequential effects should be ignored.

The requirements to contribute to this study were as follows. First, contributors were asked to submit a table containing for each experiment the inferences about each of four components of response time performance: ease, response caution, bias, and non-decision time. For each decision, as for each experiment, we gave contributors the option to submit a degree of confidence in their inference.^6^ Second, contributors were asked to submit a description of their methods. This description of method was asked to meet a reasonable level of reproducibility, describing at least: 1) outlier removal procedures, 2) the applied mathematical model (if one was used), 3) the method of estimation (if applicable), and 4) the rules applied to draw inferences. Finally, contributors were asked to submit a short summary of their method. Some of the descriptions provided by the experts required further clarification, and these discussions were carried out via email. All contributors in this project are naturally included as co-authors.

## Contributed Methods of Analyses

Table 3 summarizes the different approaches contributed by each contributor or team of contributors. In what follows, we give a relatively coarse overview of the different approaches used. A full description of the methods used is given by the authors themselves in the supplementary materials that are available on the open science framework (https://osf.io/ktuy7/).

**Table 3:** Methods used by contributors

Contributors	Code	Model	Estimation inference	
Grasman	GR	Simple diffusion	EZ2	E (Quade test on ind.)
Krypotos & Wiecki	KW	Simple diffusion	HB	E (Population post.)
van Ravenzwaaij	RA	Simple diffusion	HB	E (Bayesian *t* test on pop. post.)
Vandekerckhove & Kupitz	VK	Simple diffusion	HB	M (Model indicator parameter)
White	WH	Simple diffusion	χ^2^	E (Bayesian *t* test over ind.)
Hawkins	HA	Full diffusion^1^	HB	E (Population post.)
Leite	LE	Full diffusion	χ^2^	H (Parameter estimates)
Starns	ST	Full diffusion^1^	χ^2^	E (Bayesian *t* test over ind.)
Vandekerckhove	VA	Full diffusion^1^	ML^2^	M (Wald test)
Voss & Lerche	VL	Full diffusion	KS	E (Frequentist *t* test over ind.)
Annis & Palmeri	AP	LBA	HB	M+E (wAIC + Population post.)^3^
Cassey & Logan	CL	LBA	HB	E (Population post.)^3^
Lin & Heathcote	LH	LBA^4^	ML	M+E (AIC/BIC + ANOVA)
Trueblood & Holmes Visser	TH	LBA^5^	HB	E (Population post.)
Visser	VI	LBA	ML	M (Stepwise regression)
Evans & Brown van Maanen	EB	–	–	H (Summary Statistics)
van Maanen	MA	–	–	H (Summary statistics)

HB = Hierarchical Bayes; *χ*^2^ = chi-squared; ML = maximum likelihood; EZ2 = method of moments estimation, as implemented in EZ2; KS = Kolmogorov–Smirnov; E = estimate-based; M = model selection; H = heuristic based; Pop = population; Post = posterior; Ind = individuals. ^1^Variability parameters fixed across conditions. ^2^Data treated as one participant. ^3^Assumed just one manipulation per experiment, unless extremely strong evidence otherwise. ^4^Both LBA and full diffusion model were fit, but the best fitting model was used, and this was always LBA. ^5^Bias in accumulation rate parameters. Modelers AP, CL, ST, VI, and KW were allowed 2 extra weeks after the initial deadline to hand in their inferences

### Model Choice

Contributors were roughly equally divided among three options - the Full Diffusion model, in which all of the between-trial variability parameters are estimated; the Simple Diffusion model, containing no between-trial variability parameters, and the LBA model. However, note that even though different contributors may have used a particular class of model (e.g., the LBA), there were differences in the exact instantiation of the model. For example, some of the contributors who used the Full Diffusion model chose to fix the between-trial variability parameters across the two conditions of each experiment. Contributors using the LBA also differed in the way that they parameterized the model. Finally, note that two contributors (EB and MA) used no computational model, and are indicated in Table 3 by a "-". Both teams of contributors using heuristic methods based their inferences on summary statistics, but their choices were driven by known behaviors of evidence-accumulation models.

### Estimation Type

Of those who fit computational models, about half of the contributors estimated the parameters of the model using Hierarchical Bayesian methods, wherein posterior distributions for population-level parameters were inferred. The other half of contributors fit their models separately to each of the 20 individual participants using a range of methods, including maximum likelihood, chi-squared, method of moments, and Kolmogorov-Smirnov estimation.

### Inference

There was a wide range of methods used for drawing inferences about the theoretical differences across conditions. We encourage readers to examine each of the authors’ own descriptions of their methods, but we attempt to summarize them as follows. There were roughly three approaches: estimate-based, model selection, and heuristic-based methods (or some combination thereof). In the "estimate-based" approach, the contributors used differences in the estimates of model parameters to draw inferences about differences across conditions. Most contributors had a unique way of doing their estimate-based inference, but the most popular approach was to subject the individual-participant parameter estimates to a hypothesis test (Frequentist or Bayesian). The groups who had used Hierarchical Bayes to estimate population-level parameter values generally used the posterior distribution of differences across conditions to draw their inferences, but differed on their exact method. Other contributors used model selection, such as a model-indicator parameter in a Hierarchical Bayesian model, Backwards stepwise regression, or a hybrid between estimate-based and model selection such as AIC, BIC, or wAIC combined with hypothesis tests. Finally, a number of groups used Heuristic-based approaches, based on either estimated parameters, or a range of summary statistics calculated from the raw data. For example, if the proportion of correct responses differed by more than 5% between two conditions, then they were assumed to differ in ease.

## Results

The first result worthy of mention is that the 17 teams of contributors utilized 17 unique procedures for drawing their inferences. That is, despite all attempting to solve the same problem, no two groups chose identical approaches. This result highlights the garden of forking paths that cognitive modelers face.

**Fig. 2:**
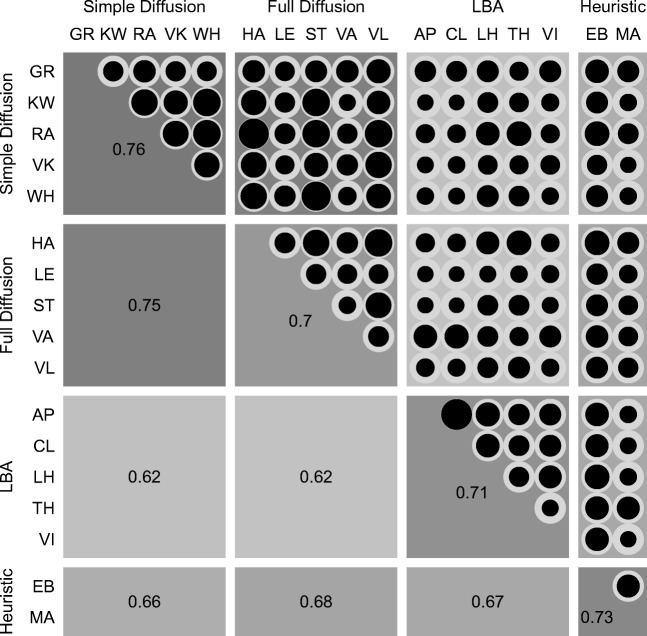
A visualization of the agreement between the different methods used. The radius of the black circles relative to the lighter colored background circles in the upper right of the matrix reflects the proportion of inferences shared between a pair of methods. The shade of the box underlying each set of points, and the numbers in the lower left of the matrix, depict the average of the proportion of shared inferences in each section. For example, the average proportion of shared inferences between all LBA and all simple diffusion models was 0.62

The complete set of inferences made by each team of contributors is given in full in Table 5. The column labelled True shows, for each experiment, for each parameter, which, if any, manipulation was made. Under each team of contributors (columns), the colored letters indicate the effect inferred by each modeling group. Inferences that are green are in line with the "True" manipulation, blue inferences indicate misses, orange inferences reflect false alarms, and black inferences are cases in which an effect was inferred in the wrong direction. Looking at Table 5, we see that many of the methods seem to make the same inferences, though there are also substantial differences. Figure 2 depicts the overall level of agreement between the inferences made by any pair of methods. The size of the circles in the upper diagonal of the matrix grows larger as the two methods yielded more similar inferences. For example, AP and CL differed only in terms of one inference (out of 56), and so the circle in that cell of the matrix is large.

The most notable pattern to come out of Figure 2 is that we see a lot of agreement within model classes. Diffusion models yielded similar inferences to one another, as did the LBA models. However, there is less agreement between the inferences coming from LBA and diffusion models. For example, the inferences from VL were similar to other diffusion model analyses, but were less consistent with the LBA or Heuristic methods. On a positive note, it is encouraging to see that although the models did sometimes reach different conclusions, there is some consensus in the inferences drawn. At worst, the overall agreement between model classes is 62%, indicating that even the methods that disagree most do complement each other reasonably well.

In what follows, we will unpack the reasons behind the patterns highlighted in Figure 2. We will focus on the performance of the different model classes, since we could identify clear differences between the patterns of inferences yielded by the different models. We did also look for systematic, aggregate-level influences of estimation or inference method, but could identify no strong, systematic effects. Any further attempt to look at select cases were compromised by the very many options available to researchers. Naturally, since the teams of contributors could select their own approach, we were unable to eliminate potential confounds between model choice, estimation, and inference method.

We begin with the analysis that was originally planned for this project, where we assume a selective influence of our experimental manipulations on the participants’ behavior - for example, speed- and accuracy-emphasis instructions should influence only response caution. However, we also present the results of two alternative analyses that were based on alternative assumptions about what is the true effect of our manipulations. These alternative analyses were based on emails that we received from contributors before they submitted their inferences. As such, we note that these analyses are not entirely exploratory.

### Planned Analysis of Validity

**Fig. 3:**
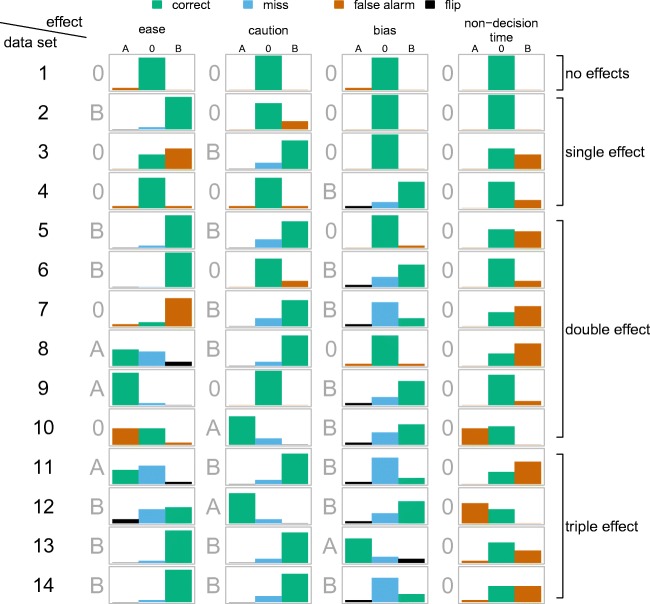
A summary of the inferences of all methods of analyses for all data sets. Grey letters in front of each box show for each data set (1-14) and for each component (ease, caution, bias, ndt), which condition (A, B, or 0: none of both) was manipulated to have a higher value on that component. Bars indicate how many methods concluded for each of the options A, 0, and B. See text for details

We start by considering the accuracy of the inferences under a selective influence assumption, in which difficulty affects ease, emphasis instructions affect caution, base-rates affect bias, and non-decision time was not manipulated in our experiments. The aggregate performance of all inferences is presented in Figure 3. The figure should be read as follows: Each row represents one of the 14 two-condition experiments. The four columns represent the four components of the response time task performance about which the collaborators drew their inferences. The grey letter to the left of each box shows the manipulated effect: an A indicates that condition A was manipulated to have a higher value than condition B on the component concerned; a B indicates that condition B had a higher value than condition A; a 0 indicates that both conditions had the same value. In other words, the grey letter indicates the "correct" inference. The size and location of the colored bars within the grey box indicate how many methods concluded in favor of inference A, 0, and B. The color of the bars indicate whether procedures concluded "correctly" (green), missed a manipulated effect (blue), detected an effect that was not manipulated (orange) or flipped the direction of a manipulated effect (black). Note that for each experiment, the largest bar thus indicates the inference made by the majority of approaches. This majority inference is not always the same as the "correct" inference.

There is a lot of green in Figure 3, indicating that a large share of the inferences made by the contributors accurately reflect the manipulated effects. Importantly, in most cases there is a clear majority conclusion. Such agreement between methods is reassuring, given the issue of researcher degrees of freedom. That is, regardless of the choice of model, estimation method, or approach to inference, the conclusions drawn from the models tend to overlap substantially.

Figure 3 also reveals a fair number of incorrect inferences. Some of the erroneous inferences in Figure 3 are common to all methods. For example, the vast majority of methods inferred an effect of ease in Data Set 7, even though we did not make such a manipulation. In other cases, the incorrect inferences are not consensual. For example, in Data Set 7 the different contributors do not unanimously agree on whether there is a difference in non-decision time between the two conditions. The majority of what follows is an exploration of the systematic errors made by the different methods.

The overall accuracy of the inferences made by the teams of contributors are reported in Table 4. We now focus on the section labeled ‘original’, which reports the accuracy of the inferences when we assume selective influence. The method that performed best, according to the proportion of correct inferences, was submitted by GR (EZ2 estimation of simple diffusion model, 84% correct inferences). Out of 56 possible inferences, EZ2 yielded only 4 misses and 5 false alarms. Note that the overall accuracy of the EZ2 approach was more than two standard deviations better than the overall, average accuracy of 71%.

**Table 4:** Summary statistics for the quality of inferences drawn using each method

key		Simple diffusion					Full Diffusion					LBA					No Model	
		GR	KW	RA	VK	WH	HA	LE	ST	VA	VL	AP	CL	LH	TH	VI	EB	MA
planned	Correct	0.84	0.73	0.73	0.66	0.75	0.73	0.71	0.75	0.77	0.71	0.66	0.64	0.70	0.62	0.68	0.77	0.70
	Miss	0.07	0	0.07	0.12	0.04	0.09	0.05	0	0.20	0.09	0.29	0.30	0.12	0.23	0.07	0.12	0.20
	FA	0.09	0.25	0.20	0.21	0.21	0.18	0.20	0.25	0.04	0.20	0.05	0.05	0.12	0.14	0.11	0.11	0.09
alternative 1	Correct	0.80	0.82	0.80	0.66	0.82	0.77	0.75	0.82	0.75	0.73	0.75	0.73	0.77	0.77	0.77	0.91	0.77
	Miss	0.11	0	0.07	0.16	0.05	0.09	0.05	0	0.20	0.11	0.25	0.27	0.11	0.16	0.07	0.07	0.18
	FA	0.09	0.16	0.14	0.18	0.14	0.14	0.11	0.18	0.05	0.16	0	0	0.09	0.07	0.07	0.02	0.05
Alternative 2	Correct	0.68	0.86	0.89	0.82	0.91	0.89	0.70	0.91	0.64	0.84	0.50	0.48	0.64	0.68	0.52	0.68	0.64
	Miss	0.23	0.02	0.07	0.12	0.04	0.09	0.12	0	0.34	0.11	0.45	0.46	0.23	0.29	0.23	0.25	0.30
	FA	0.09	0.11	0.04	0.05	0.05	0.02	0.11	0.09	0.02	0.05	0.05	0.05	0.07	0.04	0.11	0.07	0.04
No ndt	Correct	0.86	0.83	0.86	0.76	0.88	0.86	0.74	0.93	0.71	0.86	0.55	0.52	0.67	0.64	0.57	0.74	0.71

Statistics are shown for three different scoring keys (Planned: assuming selective influence; Alternative 1: assuming caution manipulations affected also ease; Alternative 2: assuming caution manipulations affected also nondecision time) as well as for the planned key when ignoring nondecision time inferences. Methods are sorted by the applied RT model, from left to right: simple diffusion model, full diffusion, LBA, and model free. See text for details

To get a sense of the accuracy for each model class, though potentially crude, we aggregate over all contributors using each model class. Averaging the accuracy of all contributors that used the full diffusion model, we observe that 73% of their inferences were correct. The simple diffusion model performed similarly, with an average accuracy of 74%. The model-free approaches also performed relatively well (74% correct). Only the inferences based on LBA models were noticeably worse than average (66%), however, the LBA model did yield fewer false alarms (9.4%) than the simple and full diffusion model analyses (17.4% and 19.2%, respectively).^7^

The different models were systematic in the types of errors they produced. Consider first the incorrect inferences of the diffusion model. Whenever emphasis instructions were manipulated, the diffusion model incorrectly identified differences in non-decision time (cf. Voss et al., 2004 and Arnold et al., 2015). For illustration, consider Data Set 3 in Figure 3. Here, only caution was manipulated, and yet many methods inferred a manipulation of non-decision time. Though not clear from Figure 3, also looking at Table 5 indicates that all of these errors come about due to diffusion models. The same pattern is repeated in Data Sets 5, 7, 8, and 10-14: whenever caution was larger in one condition, the diffusion models tend to infer that non-decision time was also larger. Note that the EZ2 diffusion model was less likely to infer changes in non-decision time, and the lack of such errors is responsible for its superior performance.

**Table 5:**
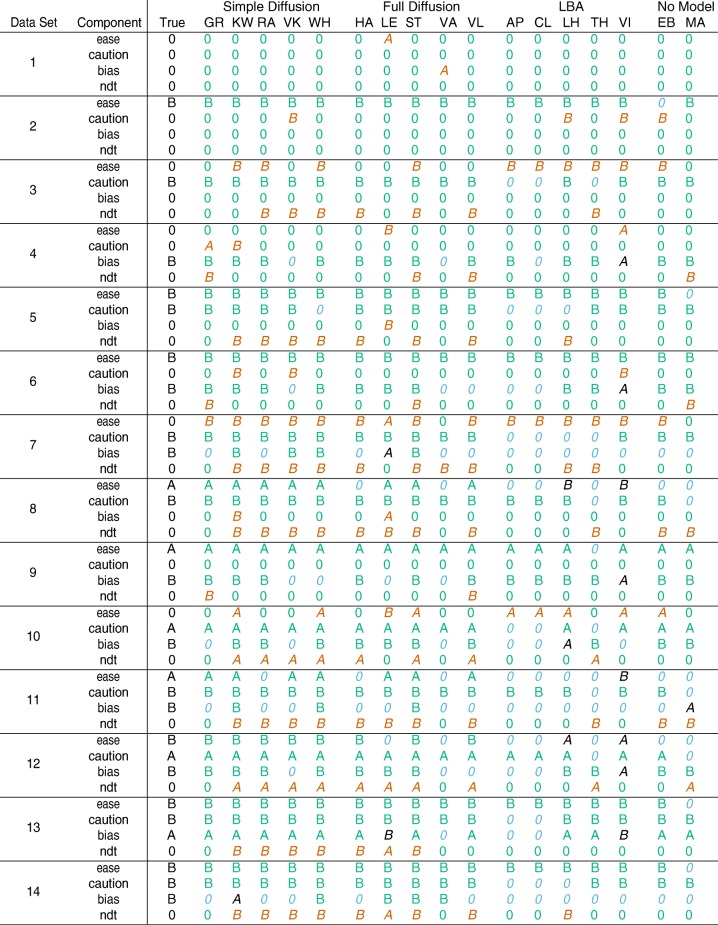
Performance of the different methods

Column “True” shows for each data set, for each component (ease, caution, bias, ndt), which condition (A, B, or 0: none of both) was manipulated to have a higher value on that component. Colored letters indicate inferences made by the analysts. Green letters indicate correct inferences, blue misses, orange false alarms, black cases where there direction of the effect was flipped. Methods are sorted by the applied RT model, from left to right: simple diffusion model, full diffusion, LBA, and model–free

The LBA model also made systematic errors in inference. The incorrect inferences made by LBA also relate to the manipulation of caution. Unlike the diffusion model, the LBA model often infers that manipulations of speed emphasis affect the ease of processing. For example, looking at Data Set 3 in Figure 3, we see although only caution was manipulated, many models inferred that ease was also manipulated. This issue is also present in Data Sets 7, 8, and 10-12, and in almost all cases, these incorrect inferences are produced by the LBA-based analyses.

The final source of systematic errors in inference are common to both LBA- and diffusion-based analyses, and relate to bias. We see in Data Sets 7, 11, and 14, that the bias manipulation is not detected by the majority of approaches. Unique to these experiments, both caution and bias were manipulated in the same direction. Returning to Table 2, we see that the data used to create these particular data sets involved a contrast between speed emphasis with no bias, and an accuracy emphasis condition with bias. Now, recall that our behavioral manipulation checks on hit and false alarm rates revealed no effect of the bias manipulation in the accuracy-emphasis condition. Therefore, the most likely reason that the models fail to detect a manipulation of bias in Data Sets 7, 11, and 14, is simply because the effect is not present in the data. It is worth noting explicitly that we expect no other issues with bias manipulations in any of the other data sets. In all other experiments in which bias was manipulated, our analysis of hit and false alarm rates suggested that there was a behavioral effect for the models to detect.

#### Post-hoc Analysis: Excluding non-decision time

Before we turn to the alternative methods for scoring the contributors’ inferences, we first report an interesting post-hoc analysis of our results. If we simply exclude the inferences about non-decision time when assessing the accuracy of the different approaches, we see a dramatic increase in the performance of methods using the diffusion model. Looking at the final row of Table 4, we see that the full diffusion model used by ST draws correct inferences in 93% of cases. In fact, almost all diffusion model analyses are accurate (> 80%) once non-decision inferences are ignored. The EZ2 diffusion model also fairs very well under this alternative scoring technique (86%). Of course, we chose to perform this analysis after having seen the data, and so this analysis is itself subject to issues with researcher degrees of freedom. Further, this analysis is only possible because we did not intend to manipulate non-decision time. As such, the results based on this post-hoc analysis should be taken with a grain of salt.

### Alternative Analysis I: Caution and Ease

A recent paper by Rae, Heathcote, Donkin, Averell, and Brown (2014) provided both empirical and model-based evidence that manipulations of speed- and accuracy-emphasis may influence both caution and the rate of evidence accumulation (see also Heitz & Schall, 2012; Starns, Ratcliff, & McKoon, 2012; Vandekerckhove, Tuerlinckx, & Lee, 2008).^8^ Intuitively, when emphasizing accuracy, participants may also try harder to do the task, in addition to collecting more evidence before responding. As such, we now consider the accuracy of the inferences made by the teams of contributors when we assume that emphasis manipulations should affect both caution and ease of processing. For example, we manipulated only speed emphasis in Data Set 3, but under this alternative scoring scheme we now consider the correct inference to be that both ease and caution was larger in condition B. Note that in Data Sets 8, 11, and 12, the manipulations of caution and ease are in opposite directions, making it difficult to rescore those conditions, and so we exclude the analysis of these experiments in this section. The second section of Table 4 reports the accuracy of the inferences under this alternative scoring method. First of all, note that the average performance of the methods increases from 75% under the original scoring, excluding Data Sets 8, 11, and 12, to 85% under these alternative scoring rules. Indeed, all models appear to do well under this scoring method (LBA: 76%, simple diffusion: 78%, and full diffusion: 77%; model-free methods: 84%). Such an improvement in performance could be consensual evidence from the models to indicate that emphasis instructions do indeed influence both caution and ease. The most accurate inferences under this alternative scoring scheme is the model-free method used by EB, whose inferences were accurate in 91% of cases. It is worth noting that their model-free method is based on years of experience with model-based methods. Interestingly, the EZ2 model remains among the better performers under this alternative scoring method, maintaining an accuracy of 80%. It should not be surprising that the LBA model performs better under this scoring scheme, since the major failing of the LBA model under the original scoring rule was that it would detect ease manipulations when caution was manipulated.^9^

### Alternative Analysis II: Caution and Non-Decision Time

Rinkenauer, Osman, Ulrich, Müller-Gethmann, and Mattes (2004) provide neuroimaging-based evidence that manipulations of speed- and accuracy-emphasis may affect both caution and non-decision time. Intuitively, when asked to be more accurate, participants may take some additional time when making their motor response, for example, checking that the button they intend to press is appropriate. We now consider the accuracy of the contributors’ inferences based on the assumption that manipulations of speed- and accuracy-emphasis influence both caution and non-decision time. For example, in Data Set 3, it would be correct to identify that condition B showed both more caution and a slower non-decision time.

The overall accuracy of inferences in this alternative analysis is largely unchanged from the original analysis (72% and 71% for the alternative and original analyses, respectively). However, the average performance of all methods is misleading, since some methods perform much better under this alternative scoring, while other methods perform much worse. The simple diffusion model performs very well under this particular coding scheme, with an average performance of 83%. The full diffusion model also performs well, with an average accuracy of 78%. The model-free and LBA-based analyses, on the other hand, perform considerably worse under this alternative scoring (66% and 56%, respectively). It is worth noting that, unlike in the previous analyses, the EZ2 model performs relatively poorly under this alternative analysis (68% accuracy). Again, this pattern of results is not surprising, but simply reflects the advantage offered to models that detect non-decision time differences whenever caution is manipulated (i.e., the diffusion models, but not EZ2 or LBA models).

## Discussion

### Summary and Recommendations

In this project, we studied the validity of inferences that we draw from response time data when we apply various models. For this goal, seventeen teams of response time experts analyzed the data from 14 two-condition experiments. For each data set, the experts were asked to infer which of four potential factors were manipulated between conditions. Of foremost importance is that inferences of this kind are not possible without a cognitive model. The contributors who used Heuristic methods also based their methods on years of experience with such models.

The first result worthy of mention is that no two teams spontaneously adopted the exact same approach to answering this question. Rather, we saw a variety of different models, estimation techniques, and inference methods across the different groups. However, despite the variety of methods, in general, we saw that the modeling teams reached a strong consensus over which manipulations we made, even when the inferences were not "correct". Overall, regardless of the scoring method we used, inferences from the diffusion model tended to be accurate. Of the model-based analyses, the simple and full diffusion models have the highest accuracy across all four alternative scorings that we considered. Further, there appears to be considerable agreement between the inferences made by the different diffusion model analyses. Given the similarity between inferences drawn from the diffusion model, it appears that the conclusions drawn are robust to many of the choices available to researchers. Many methods using the diffusion model detected effects on non-decision time where response caution was manipulated. This result is consistent with empirical evidence suggesting that manipulations to increase caution do also increase non-decision time (Rinkenauer et al., 2004), as well as previous validation studies (Voss et al., 2004). As such, we may want to apply a more careful interpretation of results such as those in Dutilh, Vandekerckhove, Tuerlinckx, and Wagenmakers (2009), in which both boundary separation and non-decision time were found to decrease with practice on a task. A more conservative interpretation of these results may be that only caution was changing with practice, and the effect manifested in both boundary separation and non-decision time parameters. Some of this confusion may also be a result of the strict division between encoding, motor response, and the evidence accumulation process made by current response time models.

Both simple and full diffusion models tended to provide robust and valid inferences. Therefore, we do not find any evidence that the additional assumptions of the full diffusion model improve inferences about differences in the core latent variables of interest - ease, caution, bias, and non-decision time (see also van Ravenzwaaij, Donkin, & Vandekerckhove, 2017). Similarly, we find that easy-to-implement estimation methods, such as the EZ2 method, tend to provide inferences that are as valid as the more complex estimation techniques. For those lacking the computational expertise to implement more complex approaches, the EZ2 method may be a suitable method for making inferences about processes giving rise to response time data (we hope, on the way to learning how to apply the models more generally).

We observed that, under assumptions of selective influence, the EZ2 method outperforms other methods that estimate the parameters of the simple diffusion model (cf. Arnold et al., 2015). This benefit likely comes about because the EZ2 method bases its parameter estimates on means and variances that are calculated over the full distribution of response times. Alternative methods estimate their parameters through more variable statistics, such as the minimum response time (or the 10th percentile of the response time distribution), and so are more likely to infer differences in parameters across conditions. The use of statistics estimated with smaller variance might also partly underlie the relative success of the model-inspired, but heuristic approach followed by contributors EB, who based their inferences largely on median RT and accuracy.

The LBA model yielded accurate inferences under the first alternative scoring rule, where emphasis instructions were assumed to influence both caution and ease. Given that all models performed well under such a scoring scheme, it could be that this set of assumptions best reflects the true state of the world. That is, it may be that manipulating speed- and accuracy-emphasis really affects both caution and accumulation rate. This result is also in line with earlier findings, as described above (e.g., Arnold et al., 2015; Rae et al., 2014; Starns et al., 2012; Vandekerckhove et al., 2008), but raises the question of whether and how response caution can be manipulated selectively.

Systematic differences between inferences based on the various estimation or inference methods were difficult to extract. As such, no strong recommendations come out of our study. We recommend that choices over estimation and inference be based on their suitability for the research question at hand. For example, if one has data from multiple participants each contributing only a small number of trials, then hierarchical methods may be best (e.g., Krypotos, Beckers, Kindt, & Wagenmakers, 2015). Bayesian hypothesis testing allows for evidence in favor of a null hypothesis, which is relevant when no difference between conditions is of theoretical interest. Also note that researchers can use our Tables 5 and 4 to gauge the performance of any given method. For example, having decided to use a full diffusion model, we see that using chi-squared or Kolmogorov-Smirnov estimation, and Bayesian or Frequentist t-tests all led to accurate inferences.

### Limitations of our Study

It is important to make clear the boundary conditions on the generality of our conclusions. The most limiting factor of our study is that all of the experiments had just two conditions. We chose such a simple design because it is ideal for an initial large-scale validation study. However, the extent to which our results generalize to more complex designs, studies with different numbers of trials and participants, or different tasks, remains an exciting avenue for future research. It remains to be seen whether the simple diffusion model can maintain its relatively good performance for more complex, factorial designs. We note that our entire factorial data set is available from the open science foundation archive, and so provides an open data set for researchers interested in pursuing such issues. We expect that more complex models, estimation techniques, and inference methods would do better under more complex experimental designs. For example, in a factorial experiment in which a single parameter can be mapped naturally onto changes in one factor, the parameters that explain differences in the other factor should be more readily identifiable. This notion is a common belief among response time modelers (e.g., Donkin, Brown, & Heathcote, 2011, 2009), though we are not aware of any explicit investigation into the situations in which this property holds. Therefore, in the same way that a signal detection analysis is rarely the ultimate analysis, the simple diffusion model may not always be the best available method. That said, a simple diffusion model analysis is vastly superior to any model-free method should one wish to make claims about latent processes.

Although we only studied data from a random dot motion task, we assume that our basic pattern of results holds for other simple decision-making tasks as well. Of course, this robustness remains to be tested empirically, but it is plausible that manipulations of difficulty, bias, and caution will have similar effects on behavior across paradigms such as brightness or color discrimination, judgments of numerosity, gabor patch orientation, or spatial frequency, as well as long- and short-term recognition memory.

Some of the contributors indicated that their choice of analysis was influenced by the special setting of this collaborative project. First, they had a rather short time window of 3 months to perform their analyses. Some contributors indicated that with more time, they would maybe have invested time reconsidering various model choices. Second, the comparative nature of the project may have encouraged the use of relatively novel approaches from some contributors. It is possible that under more typical settings, the range of estimation and inference methods may be less variable. The researcher degrees of freedom on display in our project may be more variable than what would be typically encountered in the literature.

For reasons outlined earlier, we chose to keep the modeling teams blind to the experimental manipulations we made. Such blinding has desirable properties for validity studies, but creates a rather difficult and maybe artificial inferential process for modelers. Indeed, because the analysts were completely blind to the manipulations, their analyses were entirely exploratory. In "real applications", where researchers have well-informed prior expectations to guide their inference, we expect inferences to be more accurate (though also likely more biased). For example, in non-blinded applications we would not often see inferences in which the direction of an effect were "flipped". Potentially, prior information about experimental manipulations can correct for some of the issues we discussed earlier. For example, in a Bayesian analysis, such information may help inferences by setting informative priors to convey sensible expectations about non-decision time. Alternatively, non-decision time parameters may be fixed across conditions in which they are not expected to change.

### Application Beyond Response Time Data Inference

Beyond this article’s direct importance for response time data analysis, this project illustrates the strength of blinded analyses in a many-lab collaboration. The blinding allows us to draw much stronger conclusions than would have been possible if the same researchers who designed the experiments had also analyzed the data. The collaborative nature allows us to draw much broader conclusions than would have been possible if only one lab had designed and applied a method of analysis. In particular for studying research questions where some answers are more desirable than others, this approach is invaluable. Even in the case of validating response time models, some results are more desirable than others, and so the blinded analysis presumably protects against bias. The inspiring study by Silberzahn et al. (2015) applied "statistical crowdsourcing" to address the question whether soccer players with a dark skin tone receive more red cards than players with a light skin tone. In such controversial cases, a blinded analysis would be superior to the original unblinded approach. For this reason, we hope that future validation studies will adopt this approach and improve the reliability and generalizability of their results.

## Conclusions

This study offers a better understanding of the validity of inferences that we draw when using cognitive models to analyze response time data. Indeed, the knowledge gained from this project will still grow when analysts apply new or improved methods to the openly available data sets and contribute the results to the open science framework project page^10^. The current results justify the increasing popularity of response time modeling techniques to a large extent. Future efforts should provide an even more solid basis for the advancement of response time modeling techniques throughout psychological science.

## Footnotes

1 Though Gomez, Perea, and Ratcliff (2013) argued that masked priming in a lexical decision task selectively influences encoding time, we do not use a lexical decision task.

2 The order of blocks was constant across participants. Although practice effects could potentially have influenced the results, various analyses can be found in the online supplementary materials (https://osf.io/ ktuy7/) that convinced us that the extended training phase and the distribution of block types over the sessions guarded against such effects.

3 All ANOVAs were performed using the BayesFactor package for R (Morey, Rouder, & Jamil, 2014). Effect sizes are *ω*^2^ values, calculated based on output from the aov function in R.

4 The code creating the data sets for the modelers accidentally contained a line deleting trials quicker than 200 ms. This resulted in the deletion of 0.16% of the trials, and so unlikely has influence on our results.

5 The analysts received the data sets in order: 4, 5, 12, 3, 13, 11, 10, 8, 6, 1, 7, 9, 2, 14.

6 We chose not to analyze the confidence ratings given by contributors, owing to the heterogeneity in responses. However, the inferences including confidence ratings are available in the supplementary materials on the project’s osf page (https://osf.io/ktuy7/).

7 Note that two of the contributors using the LBA, AP and CL, made the assumption that only one parameter would vary across the two conditions, necessarily lowering the false alarm rate. However, excluding their inferences does not change the overall conclusion that the LBA-based analyses yielded fewer false alarms. Indeed, throughout the paper, we looked at the impact of excluding AP and CL on the conclusions about model class and found it to have little impact.

8 The Rae et al. (2014) paper also found that emphasis instructions affected non-decision time, but since this was not the focus of the email that prompted this analysis, nor of the Rae et al. manuscript, we do not consider an analysis in which emphasis manipulation influences drift rate, boundary separation, and non-decision time.

9 The two alternative scoring rules are based on the assumption that the models are telling us about regularities in the world - i.e., that emphasis instructions influence both ease and boundary separation. It is also possible that such inferences are due to correlations between model parameters. For example, the estimates of accumulation rate and threshold are correlated in an LBA model, while boundary separation and non-decision time are correlated in a diffusion model. It is impossible to disentangle such issues in our data, and so this alternative interpretation of our results is worth keeping in mind.

10 https://osf.io/jy7pi/. An example of such contribution is already submitted post hoc by contributor LE, who corrected a typo, thereby improving his inferences considerably.
